# Imaging in a Rare Case of Neonatal Arterial Tortuosity Syndrome

**DOI:** 10.1055/s-0043-1775980

**Published:** 2023-10-10

**Authors:** Maria Cristina Inserra, Alessia Di Mari, Giulia Passaniti, Maria Teresa Cannizzaro, Giuliana La Rosa, Daniela Poli, Placido Gitto, Laura Patanè, Placido Romeo

**Affiliations:** 1 CAST Radiology Department, A.U.O. Policlinico “G.Rodolico-San Marco,” Catania, Italy; 2 Department of Medical Surgical Sciences and Advanced Technologies “GF Ingrassia,” University Hospital Policlinico “G. Rodolico-San Marco,” Catania, Italy; 3 CAST Division of Cardiology, A.O.U. Policlinico “G. Rodolico - San Marco,” Catania, Italy; 4 Department of Medical Surgical Sciences and Advanced Technologies “GF Ingrassia,” University Hospital Policlinico “G. Rodolico-San Marco,” Catania, Italy; 5 CCPM – Centro Cardiologico Pediatrico del Mediterraneo “Bambino Gesù” di Taormina, Italy; 6 Radiology Department of AO “San Marco,” A.U.O. Policlinico “G.Rodolico-San Marco,” Catania, Italy

**Keywords:** arterial tortuosity syndrome, SLC2A10, coarctation of the aorta, connective tissue disorder, aortic elongation sign

## Abstract

Arterial tortuosity syndrome (ATS) is a very rare autosomal recessive disorder that affects the connective tissue. The incidence of ATS is not well known and to date only 106 patients have been described in the literature. ATS affects medium and large size arteries, leading to widespread elongation and intensification of the average vessel tortuousness, responsible of several loops and kinks. Like other connective tissue disorders, ATS can present with joint laxity, hernias, pectus excavatum, scoliosis or other musculoskeletal abnormalities, and ocular defects. Due to the extreme variability of clinical symptoms and the fact that ATS has no curative management, prompt diagnosis is of tremendous importance to prevent disease-associated complications. In this situation, imaging techniques have a central role. In this study, we describe a rare case of a male newborn with tortuosity and lengthening of the main arterial and venous medium and large caliber branches with associated aortic coarctation who passed away prematurely. The finding of aortic coarctation in a newborn with ATS has rarely been described in the literature.

## Introduction

Arterial tortuosity syndrome (ATS; OMIM 208050) is a very rare autosomal recessive disorder that affects the connective tissue. It was first described in 1967 by Ertugrul. 1

The incidence of ATS is not well known and, to date, only 106 patients have been described in literature, but its incidence is supposed to be even higher due to its very large and difficult-to-identify manifestation spectrum. 2 ATS is related to mutation of the gene SLC2A10 (chromosome 20q13.2), which encodes for the glucose transporter GLUT10. The exact role of GLUT10 in the pathogenesis of the disorder remains to be fully clarified. Previous evidence revealed that GLUT10 acts as an intracellular transporter of dehydroascorbic acid that functions as a hydroxylation cofactor for prolyl and lysyl residues, which is crucial for elastin and collagen maturation. 3 Additionally, GLUT-10 deficiency alters the canonical pathway of transforming growth factor β (TGF-β), involved in the disorganization of different proteins of the extracellular matrix, which are essential for structural integrity of different connective tissues including the blood vessel walls. 2

Specifically, ATS affects medium and large size arteries, leading to widespread elongation and intensification of the average vessel tortuousness, responsible of several loops and kinks, with a predisposition to dissection and aneurysms. CoA in association with ATS is a rare condition, with only a few cases described in the literature. 4 5

It is necessary to suspect ATS in newborns who present with typical facial features (micrognathia, convex nasal ridge, hypertelorism, high palate), musculoskeletal alteration with joint laxity (76%), hernia, pectus excavatum (28%), scoliosis or other musculoskeletal abnormalities, and ocular defects, especially if, after imaging examination, an increased vascular tortuosity is evident. 5 6

The main cardiovascular findings reported in literature are aortic tortuosity (92%), tortuosity of other arteries (80%), aortic root aneurysm (16%), pulmonary artery stenosis (57%), focal stenosis of the aorta (24%), autonomic dysfunction (18%), aortic and arterial aneurysms, stenosis of the aortic valve and pulmonary arteries, and ischemic or hemorrhagic events. 5

Due to the generalized abnormalities caused by ATS, early diagnosis is of fundamental importance to try to prevent life-threatening complications.

However, the diagnostic criteria are still not well established. Therefore, the diagnostic pathway begins with a strong clinical suspect, continues with advanced imaging multimodality techniques (radiography, echocardiography, computed tomography angiography [CTA] evaluation, and/or magnetic resonance [MR] angiography) and ends with genetic research of the SLC2A10 mutation.

We describe the rare case of a newborn with typical facial features, increased skin folds, showing typical imaging results of ATS in CTA with aortic coarctation (CoA) that died about 2 months and 15 days from birth due to severe diffuse ischemia.

## Case Presentation

We present the case of a male infant, born at 37 weeks of gestation (W.G.) by urgent cesarean section for growth arrest.

The infant's birth weight was 2.250 g (3th percentile), height was 47 cm (10th percentile), and occipitofrontal circumference (OFC) was 34 cm (3th percentile).

Family history was unremarkable. The infant was first child to healthy nonconsanguineous parents originating from southern Italy.

The patient's prenatal echocardiographic study showed evidence of complex congenital heart disease.

At birth, the infant was hemodynamically stable and in spontaneous breathing and was admitted to the neonatal cardiologic intensive care unit at birth for further investigations and appropriate treatment.

**Fig. 1:**
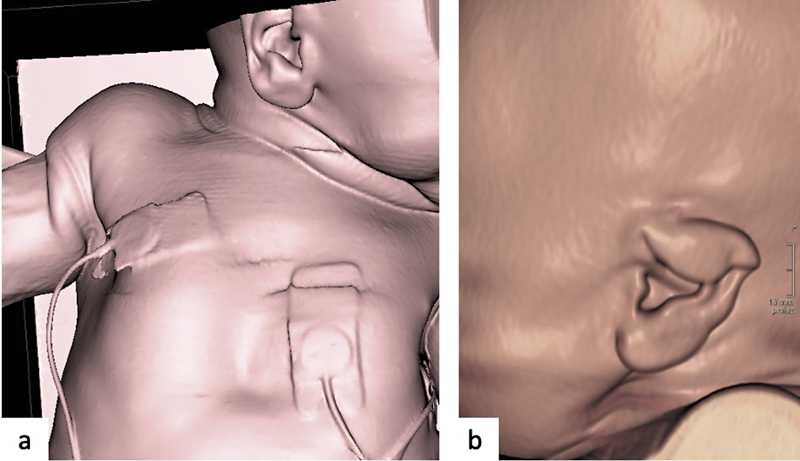
Skin laxity and helix folding. Surface-rendered three-dimensional (3D) reconstruction images showing excessive skin laxity leading to multiple redundant skin folds in the ( **a** ) axillary regions and neck with long ear and ( **b** ) helix folding.

On physical examination, the newborn presented dysmorphic facial features (wide folded ears and narrow maxilla), increased skin plication, acrocyanosis, minimal chest excavation, joint hypermobility ( Fig. 1 ), pronated hands, and flexed, twisted feet.

The cardiac auscultation displayed a continuous murmur (systolic–diastolic murmur) due to patent ductus arteriosus (PDA).

A transthoracic echocardiogram (TTE) was performed and it showed tortuosity at the origins of the great vessels that appeared elongated, with an ill-defined course, patent foramen ovale with bidirectional shunt, no left or right ventricular outflow tract obstruction, mitral valve dysplasia with mild regurgitation and mild aortic regurgitation, left ventricular hypertrophy, pulmonary branches with crossed pulmonary arteries (CPAs), without stenosis on the trunk or branches, and PDA.

The aortic arch demonstrated reduced caliber in its extremely tortuous course with evidence of significant turbulence on Doppler and increased caliber of the ascending aorta.

In view of these findings, at 4 days of life, a multidetector computed tomography (MDCT) angiography examination was performed with a multidetector CT (GE LightSpeed Plus 16 Slice) and multiplanar reconstruction (MPR). Maximal intensity projection (MIP) and three-dimensional (3D) volume-rendering virtual reality (VR) and surface-rendering (SR) reconstructions were also done.

**Fig. 2:**
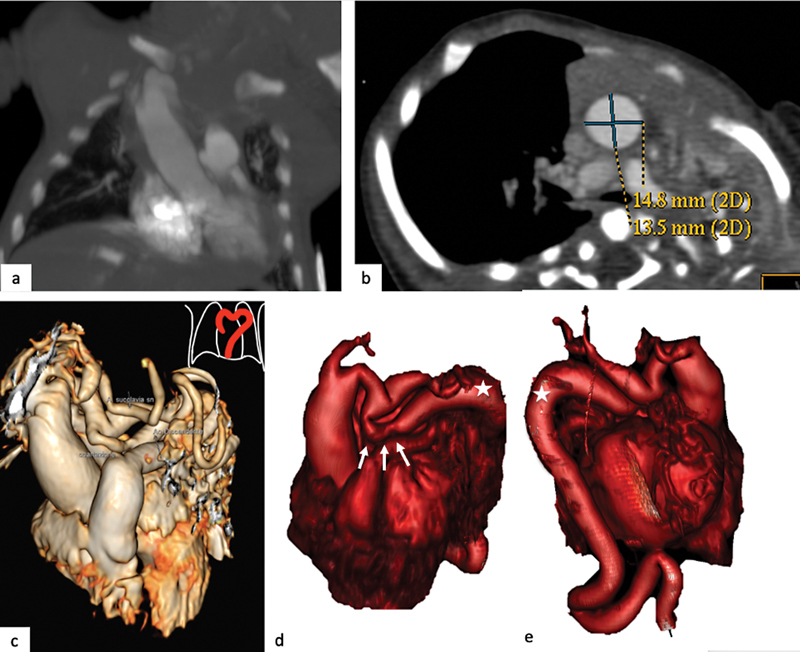
Ascending elongated aorta, quadrangular aortic arch, and aortic coarctation. ( **a** ) The coronal computed tomography (CT) image shows the ascending elongated aorta. ( **b** ) The axial CT image shows augmented size of the ascending aorta in relation to age ( *Z-* score: 5.08). ( **c** ) Three-dimensional (3D) virtual reality (VR) image shows an aortic arch with elongated appearance, tortuosity, and with focal narrowing and dilations, such as to reproduce a quadrangular appearance. ( **d,e** ) The presence of aortic coarctation ( *white arrows* ) and tortuosity of the descending aorta ( *star* ) can be observed.

The CT scan revealed widespread tortuosity, kinking, looping, coiling, and elongation of arterial and venous tree. We found an ascending aorta of augmented caliber, 14.8 mm × 13.5 mm ( *Z* -score: +5.08), which also appeared elongated ( Fig. 2 ). The aortic arch appeared elongated, with an irregular path (located to the right and higher than normal, with a quadrangular shape; Fig. 2 ).

**Fig. 3:**
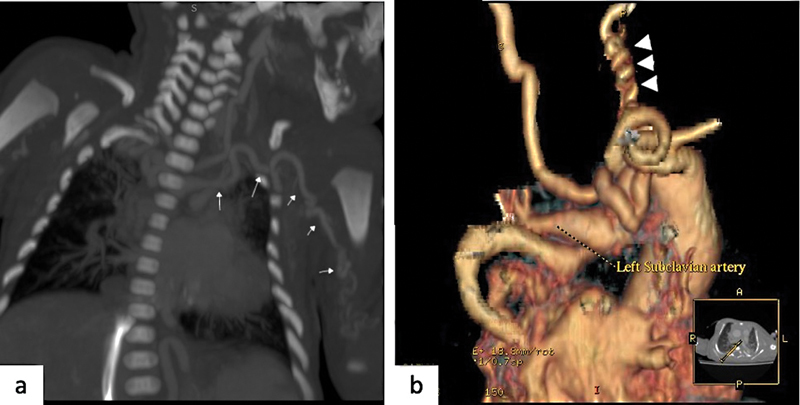
Left subclavian artery tortuosity and corkscrew appearance of left the carotid artery. ( **a** ) Coronal computed tomography (CT) image shows tortuosity with kinking and looping of left subclavian artery specially in the axillary and midarm region ( *white arrows* ). ( **b** ) Three-dimensional (3D) virtual reality (VR) image shows corkscrew appearance of the left carotid artery ( *white arrowhead* ).

**Fig. 4:**
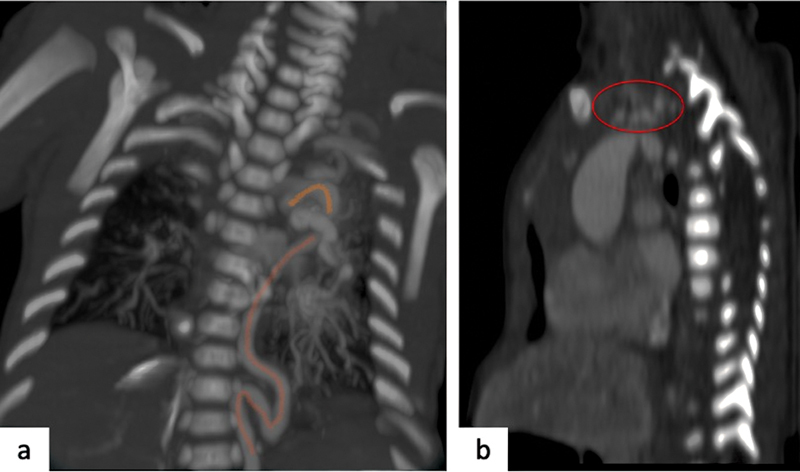
Meandering vessels sign and cluster sign. ( **a** ) Coronal computed tomography (CT) image shows “meandering vessel sign” with tortuosity of thoracic aorta and left side intrathoracic dislocation of this vessel ( *orange line* ). ( **b** ) Sagittal CT image show “cluster vessels sign” with cluster appearance of supra-aortic trunks ( *red circle* ).

**Fig. 5:**
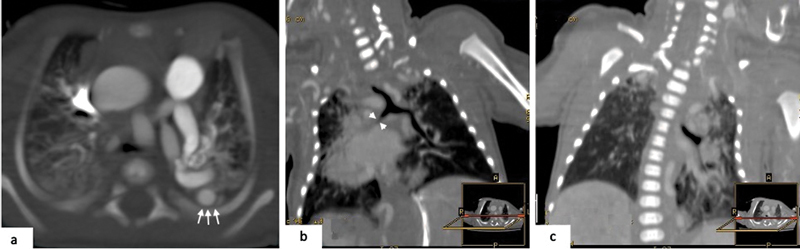
Intrathoracic course of descending aorta with collapse of lung parenchyma downstream obstruction. ( **a** ) Axial computed tomography (CT) image shows descending aorta with left intrathoracic course (white arrows). ( **b,c** ) Coronal reconstruction of contrast-enhanced CT images shows the presence of bronchial compression ( *arrowheads* ) with atelectasis of the downstream parenchyma.

The origin of the supra-aortic trunks was normal, but these vessels appeared extremely elongated and tortuous, especially the subclavian and carotid arteries, which showed a “corkscrew appearance” in the proximal and middle portions ( Fig. 3 ), with focal and multiple narrowing and widening and with progressive increase of tortuosity to the control CT. The descending aorta appeared coarctated, with abrupt narrowing at the isthmus, extended for approximately 2 cm ( Fig. 2 ). The proximal descending aorta also appeared with altered course, longer than normal, and lateralized ( Fig. 4 ), with a tendency to extend to the left hemithorax (∼3 cm from the vertebral soma in the context of the rib cage) resulting in bronchial compression and lung collapse ( Fig. 5 ); It also presented right-sided kink near the diaphragmatic hiatus ( Fig. 3 ).

**Fig. 6:**
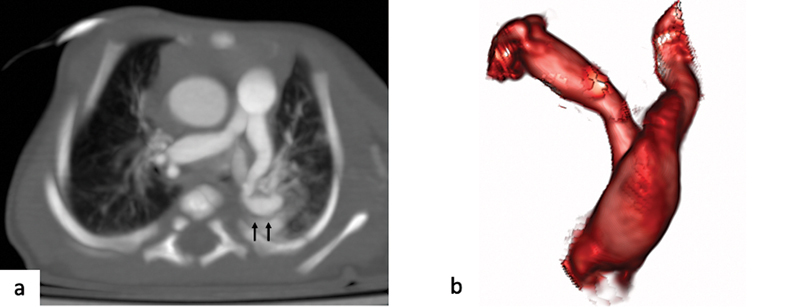
**V** sign of the pulmonary artery. ( **a** ) Axial contrast-enhanced computed tomography (CT) images with maximum intensity projection (MIP) reconstruction and ( **b** ) volume-rendered image showing elongation and early branching of pulmonary artery with a **V** -shaped configuration and winding course and segmenting narrowing and dilations ( *black arrows* ).

The main pulmonary artery appeared to be of a regular caliber ( *Z* -score: + 0,95) with early bifurcation (inverted **V** -shaped configuration at the axial CT scan) and relative elongation and narrowing of the right and left pulmonary arteries. The left pulmonary artery presented mid-distal narrowing followed by ectasia of the branch itself, which hyper-arborized the distal circles of both the pulmonary arterial districts (in the subhilar site; Fig. 6 ).

**Fig. 7:**
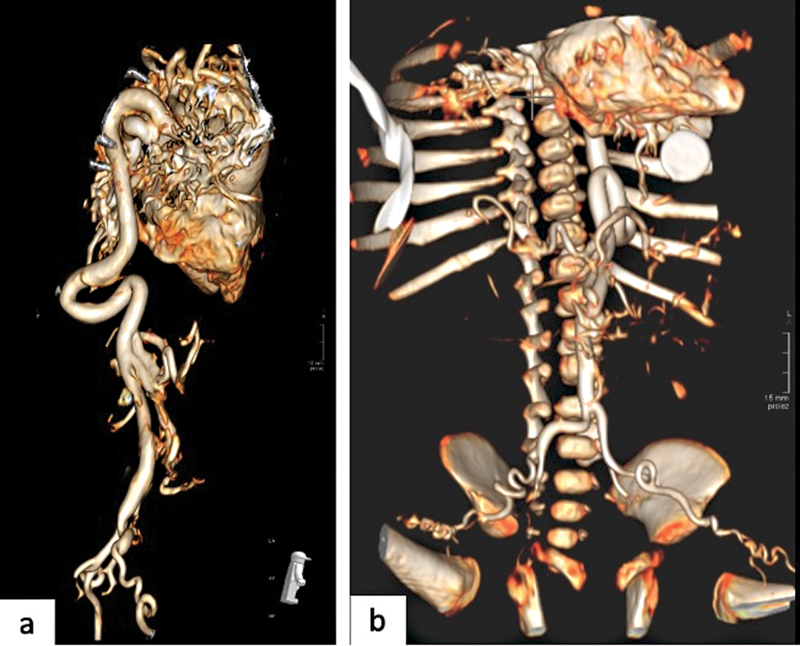
( **a,b** ) Computed tomography angiography of the chest and abdomen with three-dimensional (3D) rendered reconstruction images show tortuosity with kinking of the first tract of the abdominal aorta, and the corkscrew appearance of the common iliac arteries bilaterally and of the main visceral arteries.

The remaining medium- and small-caliber arteries and venous vessels also appeared tortuous, especially the femoral arteries bilaterally with a corkscrew appearance ( Fig. 7 ).

The circumscribed image of the plausible diaphragmatic laxity in the paracaval site with a portion of adipose tissue is also appreciated.

In relation to the CTA findings, suspicion of ATS was placed and clinical instrumental monitoring of the right superior limb and lower posteroanterior (PA) limb and SpO _2_ was performed every 4 hours.

Initial DNA analysis was performed from the DNA extracted from the peripheral blood samples from the child. Array comparative genomic hybridization (CGH) was performed to exclude Ehlers–Danlos syndrome. Based on the negative finding, the exons and intronic flanking regions of the SLC2A10 gene were amplified and direct sequencing was performed and the diagnosis of ATS was confirmed. Therefore, heparins therapy was started.

**Fig. 8:**
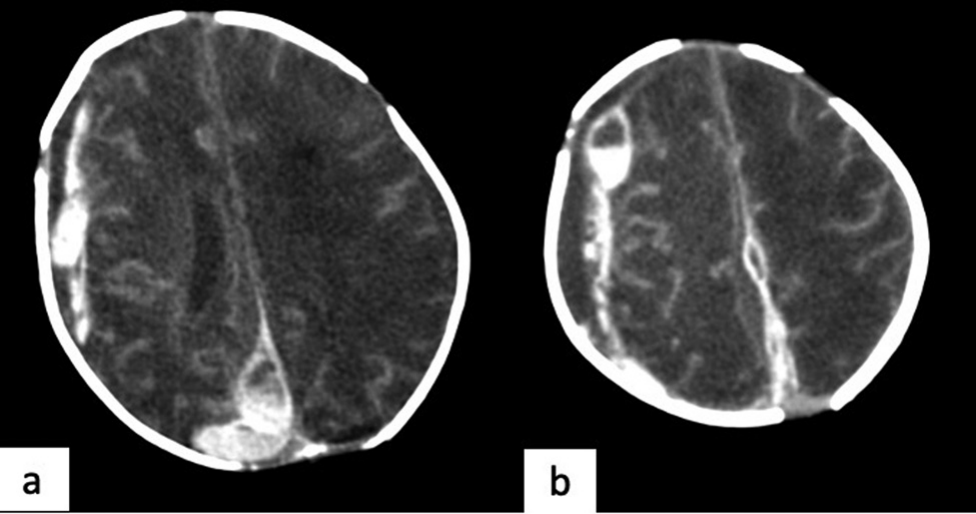
Evolutive hemorrhagic ischemic infarction. Axial computed tomography (CT) contrast images show extra-axial collection overlying ( **a** ) the right cerebral hemisphere and ( **b** ) extended on the right side of the falx.

**Fig. 9:**
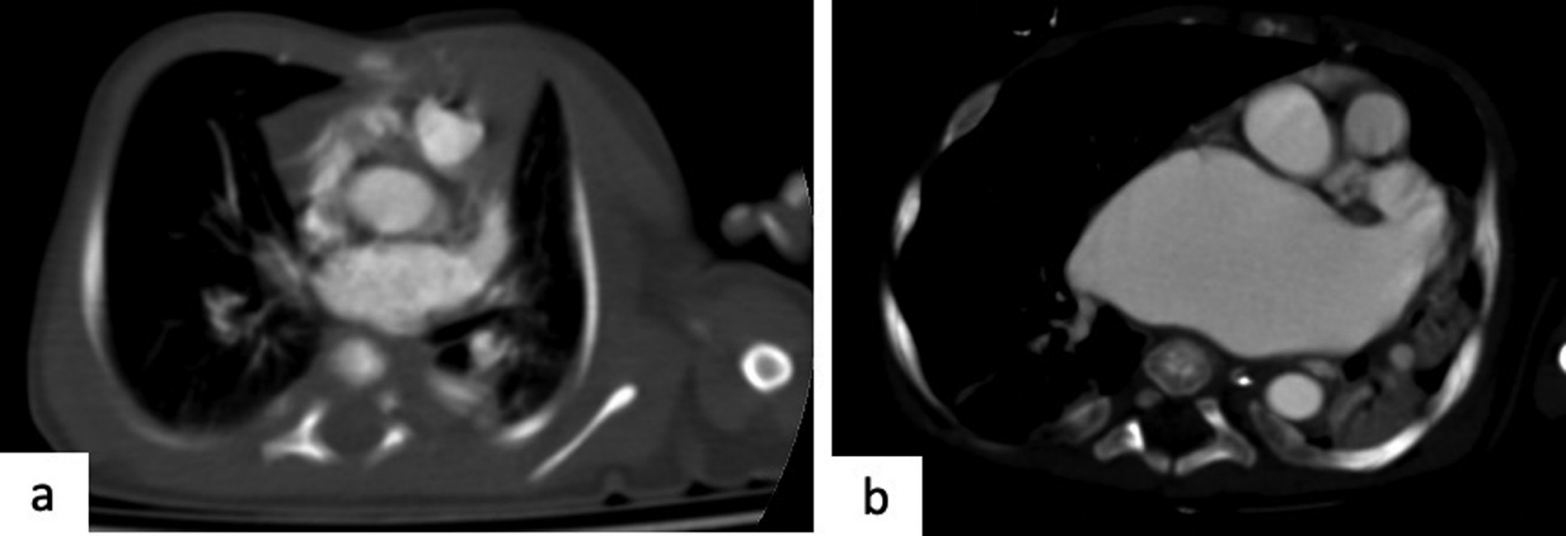
Progressive left atrial dilatation. Axial computed tomography (CT) image showing progressive expansion of the left atrium 2 months later.

Subsequently, at day 17 from birth, two CT scans were performed during the follow-up and following the detection of delayed neurological development of the child and a worsening of clinical symptoms. CT images showed a progressive increase in vessel tortuosity, encephalic ischemia ( Fig. 8 ), and marked left atrial dilation ( Fig. 9 ).

The newborn died about 2 months and 15 days from birth due to severe picture of diffuse ischemia.

## Discussion

ATS is considered a rare (<1:1,000,000 live births) autosomal recessive genetic disease, with a male-to-female ratio of 1:1. 2

The first authors who described this syndrome named it “Ehlers–Danlos syndrome with multiple pulmonary artery stenoses and tortuous systemic arteries,” underlying the similarity between these two conditions, such a pathology in fact enters in differential diagnosis with different connective diseases, for the very similar vascular, connective tissue changes and clinical presentation like Marfan's syndrome, Williams–Beuren syndrome, Ehlers–Danlos syndrome, hereditary cutis laxa syndromes, Loeys–Dietz syndrome (LDS), and lethal syndromic vasculopathy associated with a novel mutation in FBLN4; the latter are associated with greater vascular involvement. 2 3

Evidence of marked vascular tortuosity in newborn or young adults should lead to an appropriate investigation for inherited arteriopathies. It is of crucial importance to recognize specific findings associated with hereditary arteriopathies, in order to perform a targeted genetic analysis for an early final diagnosis, hoping to influence the outcome of the disease.

Imaging is of fundamental importance as it allows us to make a diagnosis, to evaluate the onset of complications, and to complete a preoperative assessment, as often such subjects need to undergo surgery. Two different quantitative methods exist for measuring arterial tortuosity. The most widely used is the tortuosity index (TI) defined as the percent ratio of calculated shortest distance between the end points divided by the actual length of the arterial segment considered. It is fundamental for the radiologist to describe the vascular anatomy, providing the index of tortuosity and a quantitative evaluation of aneurysms dilations ( *Z* -score and aortic ratio) that allows us to have a quantitative evaluation of the data found, early identification of cases of nonmanifest tortuosity, and screening of family members. It also enables better communication between the different specialists. 6 7

In his study, Bhat described the spectrum of features of ATS identified by common imaging modalities (chest X-ray, echocardiography, CTA, and MRI) that can help in the recognition of such a pathological condition that we have highlighted in our case. 8

An “aortic elongation sign” at anteroposterior (AP) chest radiography with elongation of the aorta leading to prominent aortic knuckle can be observed in a young patient. The following may also be observed: “meandering vessel sign” ( Fig. 4 ), evident at coronal CT or AP chest radiography with tortuous arteries coursing beyond the normal distribution, extending to the adjacent anatomical areas, “cluster of vessels sign” ( Fig. 4 ), expressed tortuosity originating in the great arteries leading to cluster of vessels on cross-section at CT sagittal/axial images, and “ **V** ” sign of pulmonary bifurcation (at coronal CT) or inverted “ **V** sign” of pulmonary bifurcation at axial CT expressed early bifurcation of pulmonary arteries, often with narrowing of the origin. 8

Awareness of early sign of arterial tortuosity and recognition of the described marks lead to early diagnosis of clinically asymptomatic cases of ATS. In this context, MDCT evaluation appears to be the best choice in the investigation of ATS patients.

CoA in patients with aortic ATS occurs rarely with only one report of an adult patient described in the literature. 4 It is important to identify and describe why it could benefit from surgical treatment in selected patients. In our case, the patient presented coarctation of the descending aorta, with abrupt narrowing to the isthmus, extending to approximately 2 cm, which led to a progressive increase in blood pressure in the heart chambers of the left and progressive expansion of the left atrium.

Mortality was originally estimated at 40% before the age of 5 years. 9 However, subsequent studies suggest lower death rates. 10 Still, the prognosis may be severe. The major causes of death are respiratory failure, ventricular hypertrophy resulting in generalized heart failure, myocarditis, and ischemic events. In our case, the patient had a progressive clinical deterioration with the development of pulmonary hypertension, electrocardiographic alterations consequential to the progressive atrial dilation, and subsequent ischemic events that led to early death.

## Conclusion

ATS with CoA is a very rare condition. It is crucial to maintain high diagnostic suspicion because of overlap with other connective tissue disorders.

Imaging plays a central role in the diagnosis and detection of complications. Radiologists should be aware of the imaging features of ATS for timely diagnosis. If high vascular tortuosity is found, it is critical to suspect an underlying genetic disorder. Consequently, the use of the TI helps provide a quantitative figure for this parameter. Radiologists must be familiar with the imaging features of ATS for prompt diagnosis.
